# Design of a Dual Molecular Weight Polymer Gel for Water-Shutoff Treatment in a Reservoir with Active Aquifer

**DOI:** 10.3390/polym17101399

**Published:** 2025-05-19

**Authors:** Maria Isabel Sandoval Martinez, Valeria Salgado Carabali, Andres Ramirez, Arlex Chaves-Guerrero, Samuel Muñoz Navarro

**Affiliations:** 1 Grupo de Investigación en Recobro Mejorado—GRM, Universidad Industrial de Santander, Bucaramanga 680002, Colombia; valeria2238354@correo.uis.edu.co (V.S.C.); grm@uis.edu.co (A.R.); samuel@uis.edu.co (S.M.N.); 2 Grupo de Investigación en Fenómenos Interfaciales, Reología y Simulación de Transporte—FIRST, Universidad Industrial de Santander, Bucaramanga 680002, Colombia; achavesg@uis.edu.co

**Keywords:** water shut off, soft gel, rigid gel, rheology, polyethyleneimine, polyacrylamide

## Abstract

This study presents the formulation and evaluation of a dual molecular weight polymer gel system composed of partially hydrolyzed polyacrylamide (HPAM) and crosslinked with polyethyleneimine (PEI) for water shut-off applications. A soft gel, designed for deep reservoir penetration, was formulated using 5000 ppm high-molecular-weight HPAM, while a rigid gel for near-wellbore blockage combined 5000 ppm high- and 5000 ppm low-molecular-weight HPAM. The gel system was designed at 65 °C, with an initial gelation time exceeding 8 h and viscosity values below 15 cP before gelation, ensuring ease of injection. Laboratory assessments included bottle testing, rotational and oscillatory rheological measurements, and core flooding to determine residual resistance factors (RRFs). The soft gel achieved a final strength of Grade D (low mobility), while the rigid gel reached Grade G (moderate deformability, immobile), according to Sydansk’s classification. RRF values reached 93 for the soft gel and 185 for the rigid gel, with both systems showing strong washout resistance and water shut-off efficiencies above 95%. These results demonstrate the potential of the HPAM/PEI gel system as an effective solution for conformance control in mature reservoirs with active aquifers.

## 1. Introduction

One of the major challenges in the petroleum industry is the excessive production of water [1]. Phenomena such as water coning, shifting water-oil contact (WOC), completion issues, and mechanical failures contribute to unwanted water production [2]. To address this issue, both mechanical and chemical methods have been applied in oil fields. Mechanical methods include completion tools such as packers, valves, casing replacements, and well technologies [3,4]. These techniques are known for their rapid effectiveness and relatively low cost [5,6]. However, research has shown that chemical methods, particularly those utilizing advanced polymer-based gels, tend to be more effective than mechanical approaches. These chemical treatments are especially beneficial in reservoirs with high water cuts and complex geological conditions (Joseph & Ajienka, 2010; Seifi et al., 2024; Sun & Bai, 2017) [7,8,9].

Among chemical water shut-off methods, covalently cross-linked polymer gels, particularly those formulated with organic cross-linkers such as polyethyleneimine (PEI), have emerged as one of the most effective solutions to reduce unwanted water production [10]. PEI is a low molecular weight branched polymer that offers several advantages over inorganic alternatives, including faster gelation, enhanced thermal stability, and greater tolerance to ionic strength [11,12].

Recent studies have shown that polyacrylamide (PAM) cross-linked with PEI forms a denser network compared to other systems, resulting in stronger gel structures with crosslinking reactions more controlled. In particular, PEI-crosslinked partially hydrolyzed polyacrylamide (HPAM) gels have demonstrated exceptional stability and performance, achieving permeability reductions of 95–98% even under high-salinity and high-temperature conditions [2,13]. However, its performance is highly dependent on reservoir parameters such as temperature, salinity, and pH, making it crucial to develop tailored gel formulations that adapt to these conditions for long-term effectiveness [14].

A major challenge in water shut-off design is achieving optimal gel properties before and after gelation [15]. In systems with aquifers, a successful formulation must ensure sufficient injectivity for deep reservoir penetration, while also providing effective permeability reduction, mechanical strength, and long-term stability, all while remaining cost-efficient [16]. Additionally, gels must maintain their structural integrity over time, as prolonged contact with reservoir water can lead to phenomena such as syneresis, chemical degradation, or loss of mechanical strength [17,18,19].

A promising strategy to enhance water shut-off performance is the combined injection of soft and rigid gels [20,21]. The soft gel, formulated with high molecular weight (HMW) HPAM, offers greater fluidity and deeper penetration into the reservoir [22,23]. In contrast, the rigid gel, typically based on low molecular weight (LMW) polymers, forms denser cross-links, resulting in higher mechanical strength and near-wellbore blockage [10,13,14,24]. Nevertheless, rigid gels based solely on LMW HPAM usually require higher polymer concentrations, significantly increasing treatment costs and limiting their practical application [25]. To address this limitation, a dual molecular weight polymer approach has been proposed to optimize rigid gel properties, balancing gel strength, injectivity, and cost-effectiveness, making it a viable solution for water shut-off applications in mature reservoirs [20].

Despite these advances, limited research has focused on integrated dual molecular weight HPAM systems crosslinked with PEI that combine the advantages of both gel types in a single treatment strategy. This represents a significant research gap, particularly under realistic reservoir conditions of moderate temperature and low salinity, where formulation optimization is key for both injectivity and sealing efficiency.

This study addresses this gap by formulating and evaluating a dual molecular weight HPAM gel system crosslinked with PEI. The soft gel, based on HMW HPAM, was designed for deep reservoir invasion, while the rigid gel, a combination of HMW and LMW HPAM, was intended for near-wellbore blockage. The system was optimized for a reservoir temperature of 65 °C and low salinity (1039.3 ppm), with pre-gel viscosity values below 15 cP and gelation times exceeding 8 h to ensure operational feasibility. Laboratory evaluations included bottle tests, rotational and oscillatory rheology, and core flood experiments to assess gel performance and residual resistance factors. The findings of this research will contribute to the development of a cost-effective and efficient polymer gel system, helping to minimize excess water production in mature oil fields where aquifers are present.

## 2. Materials and Methods

Materials: Anionic partially hydrolyzed polyacrylamide (HPAM) served as the base polymer, incorporating different molecular weights to develop dual gel technology with a covalent crosslinker. The soft gel was formulated using a high molecular weight polymer, while the rigid gel was created by blending high and low-molecular-weight polymers. HPAM of high-molecular-weight (FLORET TM AN 907 PG-SNF) had an average molecular weight of 7 × 10⁶ to 10 × 10⁶ Daltons, a degree of hydrolysis of 5 to 10%, and a humidity content of 9.0%. Similarly, the low-molecular-weight polymer (FLOBEADS TM AB 055 LT-SNF) had a molecular weight of 0.5 × 10⁶ Daltons, a degree of hydrolysis of 5–10%, and a humidity content of 8.0%. Polyethyleneimine (PEI) in solution with 36% active content was used as the crosslinking agent, while potassium chloride (KCl) served as the retarding agent. The formulations were prepared in a synthetic brine representative of the field brine, whose composition and properties are presented in Table 1.

Similarly, the performance evaluation of polymeric gels was carried out in sand packages from the oil formation with a low consolidation level, whose composition is shown in Table 2.

**Figure 1:**
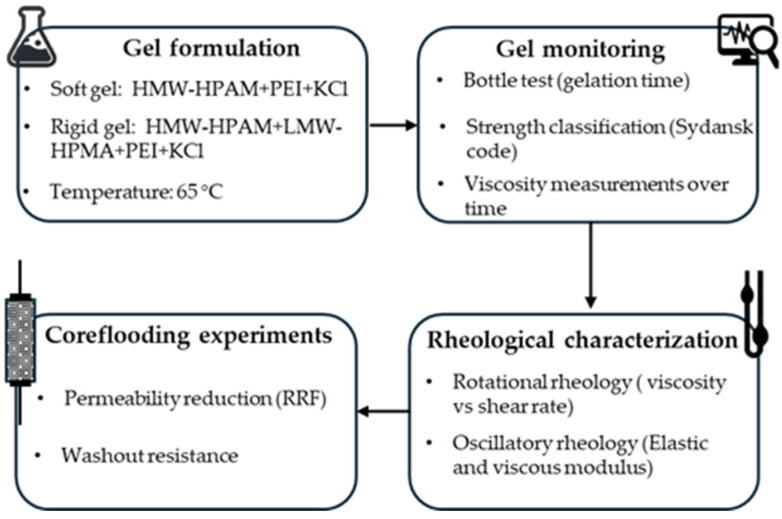
Schematic diagram of materials and procedures.

The procedures followed in this investigation are illustrated in the schematic diagram (Figure 1) and are described in detail in the subsequent sections.

Procedure for gel preparation: Gelling solutions with varying component concentrations were prepared at room temperature (25 °C). The amounts of HPAM, PEI, and synthetic were added depending on the concentrations of the polymers and the sample volume. Initially, the polymeric solution was prepared in a mechanical agitator at 500 rpm initially, then reduced to 250–300 rpm after 20 min, and maintained under these conditions for 3 h to ensure complete hydration. Afterward, the retardant agent (KCl) was added to the hydrated polymer solution and allowed to dissolve for 20 min. Finally, the crosslinking agent was gradually introduced into the polymer solution while stirring continuously at 250 rpm for approximately 20 min, ensuring homogeneity in the final gelling solution.

Strength measurements: The internationally accepted Gel Strength Coding (GSC) classification method was used to evaluate gel strength [26]. This method categorizes the strength into grades A–J according to the levels of rigidity (Table 3). The evaluation was conducted using a bottle test, where the gel was placed at the target temperature (65 °C), which corresponds to the maximum reservoir temperature. At various time intervals, the test tube was inverted, and after a few minutes, the gel state was assessed. Some codes are closely related. For instance, codes I and J exhibit similar characteristics but differ in the degree of surface vibration when subjected to impact. Likewise, the distinction between codes A and B lies in gel formation; no gel is present in code A, whereas in code B, a gel forms with a viscosity higher than that of A. Table 3 describes each code, established by Sydansk’s table (Sydansk & Argabright, 1987) [27].

This investigation aimed to formulate two types of polymeric gels: a soft gel with a strength between D and E to ensure a greater invasion radius, and a rigid gel with the highest strength, classified as grade G-H, to achieve a higher level of blocking near the wellbore.

Determination of gelation time: There are two important times in the process of gelation; the first occurs when the crosslinking process causes a significant increase in viscosity, while the second corresponds to the point at which maximum viscosity is reached due to the formation of a stable three-dimensional network. The first time was estimated based on the transition from code B to code C in Sydansk’s classification.

From these qualitative experiments with Sydansk’s code, the best formulations were selected, and they were subjected to quantitative measurements by viscosity tests. The viscosity measurements provided a more precise estimate of the moment at which the viscosity of the gelation solution begins to increase.

Rotational measurements were made in a concentric cylindrical cell with helical geometry, maintaining a 1.138 mm gap at 65 °C. This geometry was selected to have enough gelling solution (40 mL) and avoid water vaporization during the tracking time, which could affect the gelation time estimation [28].

Rheological analysis: In this investigation, an Anton Paar MCR-302 rheometer equipped with RheoCompass software (Graz, Austria) and an electrically heated temperature device (ETD) was used to take rotational and dynamic oscillatory measurements. Rotational measurements to estimate the gelling solution viscosity were carried out with cone-plate geometry, maintaining a gap of 0.1 mm at 65 °C (reservoir temperature). The viscosity curve was constructed using 40 data points, varying the shear rate between 0.1 and 1000 (1/s). All viscosity measurements were performed in duplicate to ensure the reliability of the results.

To perform dynamic oscillatory measurements, the sample of crosslinked polymer gel was made ready and aged for 24 h at 65 °C. Amplitude sweeps ranging from 0.01% to 1000% at a frequency of 1 Hz (rad/s) were conducted to determine the linear deformation region and, consequently, ascertain the strain threshold at which the gel structure fails.

Residual resistance factor estimation: After evaluating gel parameters and stability under reservoir conditions, core flooding experiments were conducted to assess the effect of the gel on absolute permeability through residual resistance factor (RRF). The RRF is the ratio of the water relative permeability of porous media before ($K_{w1}$) and after $\left(K_{w2}\right)$ gel polymeric addition. It reflects the degree of permeability decline in porous media and can be calculated using the formula provided in Equation (1).

(1) $$RRF=\frac{K_{w1\;(mD)}}{K_{w2\;(mD)}}$$

**Figure 2:**
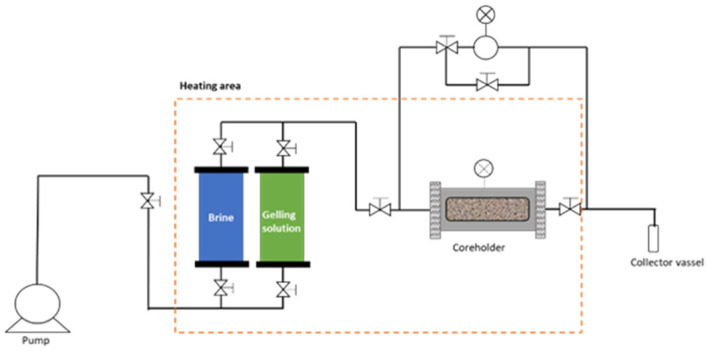
Set up test of residual resistance factor.

The core-flow experiments were conducted in a high-pressure and high-temperature core-flood assembly system (Universidad Industrial de Santander, temperature range: 25–85 °C; pressure range: 14.7–1500 psi). The equipment used an ISCO pump (model 500D, Teledyne ISCO) connected to free pistons to displace the brine and gelling solution through a porous medium. The porous medium was assembled in a steel-coreholder wired with pressure transducers and temperature sensors to monitor the behavior of these parameters during the test (Figure 2).

Sandpacks composed of formation sand were prepared within a Viton sleeve (2.54 cm in diameter, 28 cm in length). The injection volume of the gelling solution was set at 0.4 Pore Volumes (PV), and the temperature was maintained at 65 °C. The core placement time was set at twice the gelation time, as gelation in porous media can take twice as long as in bottle tests due to adsorption and dilution effects [23,29]. Once the gel formed, the formation brine was injected at 0.5 mL/min, while the pressure dropped, and the injected water volume was automatically recorded.

## 3. Results

The soft gel was formulated with a high-molecular-weight polymer at low concentrations. In contrast, the rigid gel was formulated by combining high-molecular-weight (HMW) and low-molecular-weight (LMW) polymers based on the following considerations [25]. First, polymer concentration is a key factor in achieving high gel strength. However, when HMW polymers are used, concentration is directly linked to the viscosity of the gelling solution, making it a critical parameter due to its impact on injectability. Then, an upper concentration limit must be established by the maximum viscosity that can be tolerated during pumping and gel placement (Sydansk et al., 2004) [20]. Second, while LMW polymers do not significantly increase viscosity, achieving the ideal concentration requires a large polymer quantity, making the formulation more expensive. To address this, a dual gel formulation was proposed that establishes the highest acceptable HMW concentration, defined as base loading, while incorporating the necessary amount of LMW to reach the target concentration. Additionally, this approach enhances gel rigidity, as LMW polymer molecules can occupy spaces within the HMW network, creating a more stable and reinforced gel.

### 3.1. Determination of Base Loading

**Figure 3:**
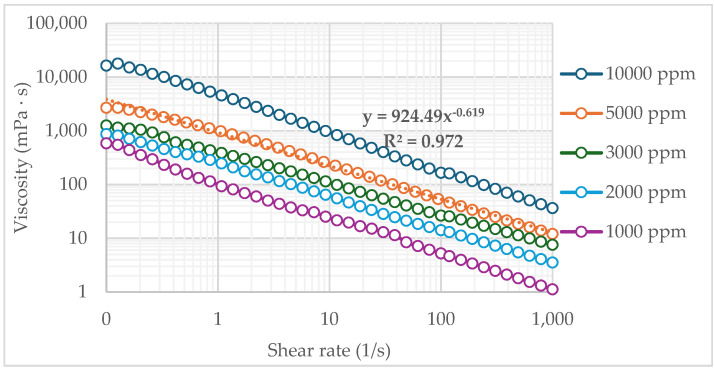
Rheograms of polymeric solutions based on HMW to estimate the base loading.

The base loading, or the highest acceptable HMW polymer concentration, was determined by analyzing its effect on the viscosity of the polymeric solution. A critical viscosity of 15 cP, in near wellbore, was considered the injection threshold, based on the formation’s fracture pressure at a maximum injection rate of 1500 bbl/day. The polymeric solution was prepared with polymer concentrations ranging from 1000 to 10,000 ppm. The viscosity curves for values of rate strain from 0.1 to 1000 1/s demonstrate that all the evaluated polymeric solutions exhibit non-Newtonian behavior, specifically a shear-thinning behavior which can be adequately represented by the Power Law model, as shown in Figure 3.

According to Figure 3, solutions with concentrations of 5000 ppm or lower exhibit viscosity values below the critical threshold at a shear rate of 1000 (1/s). Moreover, this polymeric solution follows a logarithmic linear trend with a value of consistency index (*m*) of 924.9 mPa·s^n^ and power law index (*n*) of 0.619. On the basis of this behavior, a concentration of 5000 ppm was selected as the baseline loading for the dual molecular weight polymeric gel.

### 3.2. Evaluation of Gel Strength and Gelation Time

Following the determination of the initial loading concentration, an assessment was conducted on the impact of the low-molecular-weight (LMW) concentration and the polymer-to-crosslinker ratio on rigid gels. Within these formulations, the proportion of KCl to polymer was consistently maintained at 1:1. Table 4 presents the results related to the final strengthening and initial gelation time for the properties examined, which were evaluated using the bottle test and Sydansk’s code.

The results in Table 4 indicate that polyethyleneimine, used as a crosslinker, ensures effective gelation, with gelation times exceeding 4 h. The resulting gel exhibits moderate deformability without mobility, classifying it as suitable for porous medium blocking according to the Sydansk classification. Furthermore, increasing the LMW concentration beyond 5000 ppm has a negligible effect on gel strength and gelation time. Based on these findings, a 10,000 ppm gel formulation is the most cost-effective option, and further evaluation of this rigid gel will continue.

The results indicate that increasing the polymer-to-crosslinker ratio delays the onset of gelation without affecting the final gel strength. In this study, gelation must begin after approximately 8 h, providing sufficient time for deep penetration into the reservoir. The 4:1 HPAM:PEI ratio achieved this gelation time with the lowest crosslinker concentration and was therefore selected for rheological analysis and resistance testing.

In the soft gel systems, analogous conditions were examined to those in rigid gels, focusing on the ratios of HPAM: PEI and KCl: HPAM. The evaluation was specifically directed towards polymer concentrations ranging from 2000 ppm to 8000 ppm. The results in Table 5 indicate that the reduction in polymer concentrations increases the gelation time and reduces the strength of the final gel.

From the results shown in Table 5, it can be observed that gels with polymers at concentrations below 5000 ppm are soft gels (E–D) that would allow greater invasion into the reservoir with longer gelation times. Therefore, concentrations of 5000 ppm polymer at a ratio of 4:1 with PEI are selected as polymeric gels with low mobility, as this has a viscosity close to the established (Figure 3) and reaches the expected resistance for this type of gel.

**Figure 4:**
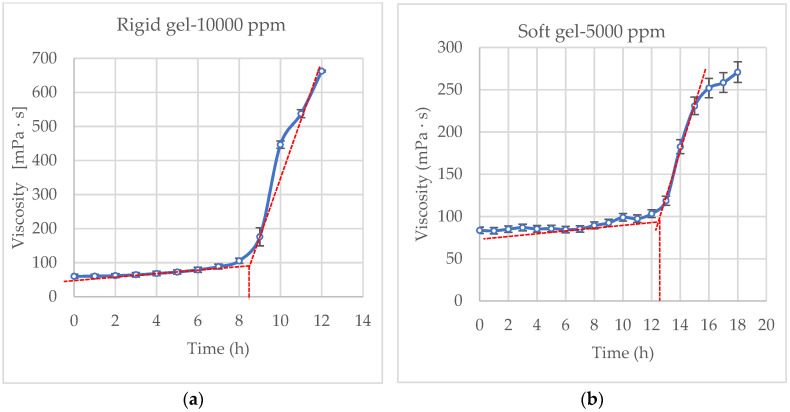
Behavior of gelling solution over time. (**a**) Rigid gel and (**b**) soft gel.

The behavior of the selected gelling solutions over time was complemented with a viscosity test for the dual rigid and soft gel, as depicted in Figure 4.

According to the research by Yu et al. (2022) [30], the gelation process can be divided into two key timepoints: the initial gelation time (IGT) and the final gelation time (FGT). The IGT refers to the moment when the viscosity of the system begins to increase noticeably, marking the transition from the slow induction stage to the fast crosslinking stage. The FGT is defined as the point at which the viscosity reaches a stable plateau, signaling the end of crosslinking and the onset of the strength stabilization stage [30].

The results of static gelation tests, including viscosity measurements, are presented in Figure 4a,b. In both formulations, viscosity initially remains nearly constant before undergoing a rapid increase, a behavior characteristic of the induction and crosslinking stages of gel formation. During the induction period, the viscosity stays below 100 cP at a shear rate of 100 s^−1^, showing only a gradual rise. This stage lasts approximately 8.6 h for the 10,000 ppm dual-polymeric gel and 13 h for the soft gel, thus identifying their respective IGTs. Following this phase, the viscosity increases sharply due to accelerated crosslinking, ultimately reaching 659 cP for the rigid gel and 270 cP for the soft gel. These trends are consistent with the gelation behavior observed in the bottle tests and Sydansk classification, thereby reinforcing the reliability of the results.

According to Figure 4, during the induction stage, both formulations exhibited similar viscosity values, as the contribution of low-molecular-weight HPAM to the initial viscosity is negligible. However, in the crosslinking stage, the dual molecular weight system (rigid gel) showed a more rapid increase in viscosity compared to the soft gel. This behavior is attributed to the greater availability of carboxylic acid functional groups for crosslinking, which is promoted by the higher overall polymer concentration. A denser polymer matrix enhances the probability of crosslinking reactions with polyethyleneimine, accelerating the gelation process and leading to a more robust gel structure [31].

### 3.3. Rheological Behavior of Gelling Solutions

**Figure 5:**
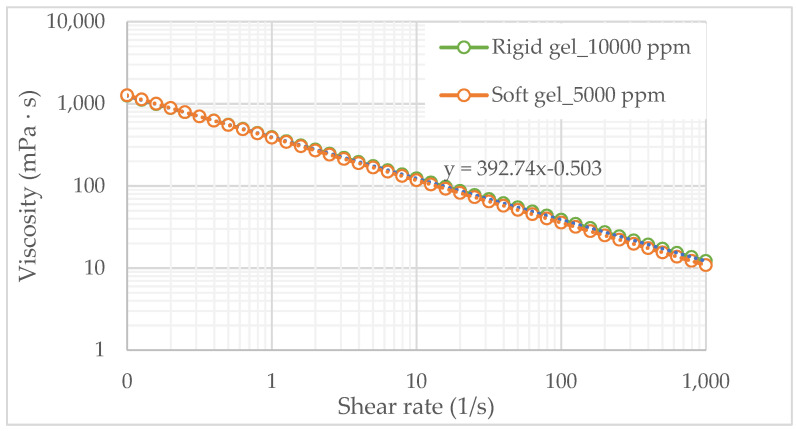
Rheology results for the dual rigid gelling solution (10,000 ppm) and soft gelling solution at a (5000 ppm) concentration with a 4:1 PEI-HPAM ratio.

Figure 5 presents the results of the viscosity curves of the dual molecular weight polymer (green line) system and soft gel (orange line) in a 4:1 PEI-HPAM ratio. As shown in Figure 5, the addition of a low-molecular-weight (LMW) polymer has a negligible impact on the viscosity of gelling solutions before the gelation process. This indicates that viscosity modifications in these systems are primarily driven by the high-molecular-weight (HMW) component and subsequent crosslinking reactions rather than by the presence of LMW polymers. However, the role of the LMW polymer becomes evident in enhancing gel strength by increasing the overall polymer concentration, contributing to the formation of a more stable and mechanically robust gel network.

Additionally, Figure 5 presents the rheological behavior of the soft gel (5000 ppm), allowing for the analysis of the differences between polymeric solutions and gelling solutions resulting from the addition of crosslinkers and retarding agents. As shown in Figure 5, gelling solutions retain pseudoplastic behavior but exhibit lower viscosity than polymeric solutions at the same concentration (Figure 3). This reduction in viscosity is primarily attributed to the presence of KCl.

Polymers such as HPAM are composed of long molecular chains with negatively charged functional groups. In aqueous solutions, these negative charges repel each other, causing the polymer chains to expand and increasing the hydrodynamic volume of the molecules, which contributes to a higher viscosity. However, when KCl is introduced into the solution as a retarder agent, potassium ions (K^+^) interact with negatively charged sites on the polymer chains, neutralizing some of these charges [32]. This phenomenon, known as electrostatic shielding, reduces the repulsion between polymer chains, allowing them to contract and adopt a more coiled structure [33]. As the polymer chains shrink and become more compact, their ability to interact with the surrounding water molecules decreases, leading to a reduction in viscosity. This effect is well documented in polymer science, where the addition of salt, especially monovalent cations like K^+^, causes polymer molecules to collapse into a more entangled but less extended conformation, reducing their hydrodynamic volume and flow resistance in solution [34].

Additionally, a distinct difference in the rheological behavior between the polymeric and soft gelling solutions at 5000 ppm is observed. Specifically, the viscosity of the gelling solution is less sensitive to the shear rate compared to the viscosity of the polymeric solution. This trend is confirmed by analyzing the power law index (n) of the power-law model that describes the rheograms of both systems (Figure 5 for soft gelling solutions and Figure 3 for polymeric solutions). This behavior is attributed to the presence of covalent crosslinkers, which reinforce the gel network and enhance its structural integrity [24]. The formation of a crosslinked three-dimensional network restricts the mobility of the polymer chain, reducing its susceptibility to shear-induced deformation. Consequently, the effect of shear on viscosity is mitigated, resulting in a more stable rheological response under varying flow conditions [30]. In contrast, when the polymer is in solution without a crosslinker, its entangled chains can realign in the direction of flow, decreasing internal resistance and lowering viscosity. This structural reinforcement explains why gelling solutions maintain a higher viscosity and greater mechanical stability under shear compared to polymer solutions.

### 3.4. Rheological Behavior of Gels

**Figure 6:**
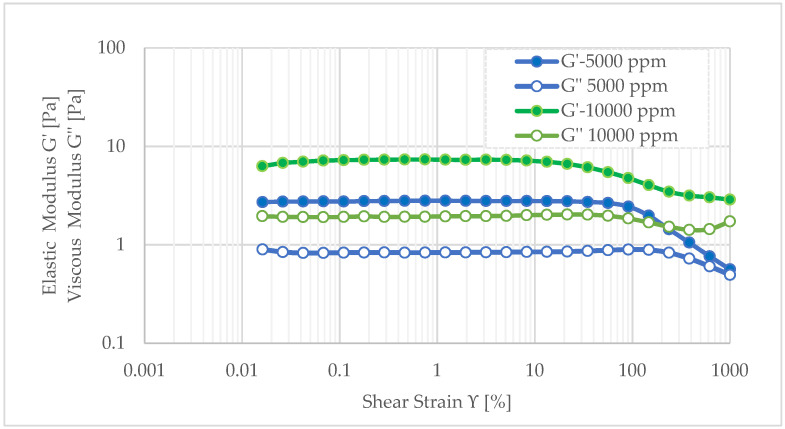
Dynamic oscillatory results for the dual rigid gel (10,000 ppm) and soft gel at a (5000 ppm) concentration with a 4:1 PEI-HPAM ratio.

According to Figure 6, it is observed that the dual polymer gel with 10,000 ppm HPAM has a constant elastic modulus around 7.2 Pa for less than 21.5% strain. Moreover, in the viscoelastic region, the value of elastic modulus (G′) is about 3.67 times more than the value of viscous modulus (G″), which indicates that the elastic modulus is dominating over the viscous modulus of gel, as is common in rigid gels. In the interval between 21.5% and 1000% strains, the value of elastic modulus is reduced to 2.87 Pa, indicating the gel is losing stiffness as strain increases. In the same interval, the viscous modulus demonstrates a slight increase, indicating an enhancement in energy dissipation. This observation suggests that the gel is transitioning from a rigid structure to a more deformable state. However, the elastic modulus remains higher than the viscous modulus, which implies that the gel retains its integrity and stretchability under very high strains, prior to any significant structural breakdown.

Conversely, the soft gel containing 5000 ppm HPAM exhibits an elastic modulus 2.58 times lower than that of the rigid gel within the viscoelastic region. Nevertheless, it endures higher strain before structural failure, maintaining stability up to 90% strain. Between 90% and 1000% strain, the elastic modulus decreases to 0.60 Pa, indicating the gel’s ability to stretch up to 900% without rupturing at 65 °C. Beyond this range, the convergence of viscous and elastic moduli signals the breakdown of the gel network. Despite its higher mobility, these properties make the soft gel a viable candidate for deep reservoir penetration and effective permeability reduction.

Similar rheological trends were reported by Pereira et al. (2021) [35] for HPAM/PEI systems at a 5:1 ratio. The authors attributed the crosslinking mechanism to the interaction between polyethyleneimine and the carboxylate groups (R–COO^−^) formed by the hydrolysis of HPAM’s amide groups (R–CONH_2_). These interactions led to the formation of a denser three-dimensional network, improving the mechanical strength of the gel, depending on polymer molecular weight, polymer, and crosslinker concentration. Furthermore, according with Yu et al. (2022) [30], gels where is added high molecular weight polymers at low concentrations present a considerable ability to block, since they can also exhibited a higher elastic modulus (G′) than viscous modulus (G″), consistent with the findings of the present study.

### 3.5. Residual Resistance Factor Estimation

**Figure 7:**
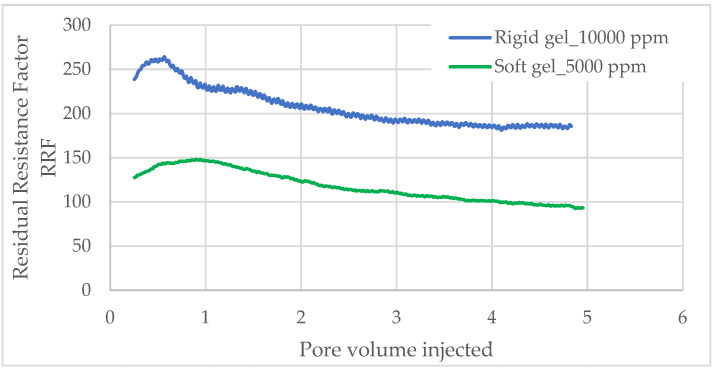
Water residual resistance factor and shut-off ratio respond as a function of the injected water volume.

The residual resistance factor (RRF) was determined for both dual rigid and soft gel systems at concentrations of 10,000 ppm and 5000 ppm, respectively (Figure 7).

Initially, for the rigid gel test, a Sandpack with an initial permeability of 8 Darcy was employed. During the subsequent water injection following gel formation, the total injected water volume was approximately 5.0 PV. The rigid gel exhibited the highest RRF of approximately 260 at an injected water volume of 0.6 PV. Beyond this point, the RRF sharply declined within the range of 0.6 to 2.5 PV. As the injected water volume increased from 2.5 to 4.8 PV, the RRF stabilized, eventually reaching a final value of around 185.

Similarly, in the soft gel, a Sandpack with an initial permeability of 7.5 Darcy was used. In this system, the gel reached its maximum Residual Resistance Factor (RRF) of 147 after nearly 1 PV of water injection. After 5.0 PV, the RRF stabilized at 93. As intended, the soft gel exhibited a lower RRF than the rigid gel, as it behaves as a weak gel that does not fully occupy the aqueous pore space.

These trends are supported by the gel strength results obtained from oscillatory rheological measurements. As the gel elasticity increases, it forms strong barriers that are more resistant to deformation. Consequently, when the rigid gel occupies the pore spaces of the reservoir rock, it remains in place and resists displacement by flowing water, resulting in higher Residual Resistance Factor (RRF) values [36,37].

According to Seright et al. (2021) [38], the soft gel typically causes modest permeability reduction, generally ranging from a factor of 2 to 100, which aligns with the results observed in this study.

The Residual Resistance Factor (RRF) depends on several properties, including the gel composition, reservoir conditions, and initial petrophysical properties. Studies have reported RRF values ranging from approximately 13.7 to over 200, relying on factors such as polymer concentration and initial permeability [29,39,40]. However, regardless of the final RRF value, Seright et al. (2021) [38] recommend that it should exceed 20 to effectively reduce permeability in water-prone zones. The RRF test results indicate that both gel systems exhibit favorable shut-off performance and washout resistance, confirming the effectiveness of the HPAM/PEI gel system in controlling water mobility, with a plugging efficiency greater than 95%.

## 4. Conclusions

This study successfully formulated two polymeric gels: a soft gel (strength D–E) for deeper reservoir penetration and a rigid gel (grade G–H) for near-wellbore blockage. The findings lead to the following key conclusions:

The dual molecular weight HPAM system (HMW + LMW) crosslinked with PEI produced a rigid gel (grade G–H) with high strength and mechanical resistance, ideal for near-wellbore blockage. The LMW fraction enabled higher polymer concentrations without raising viscosity, reinforcing the gel network. In contrast, the single HMW system (5000 ppm) formed a soft gel (grade D–E) with greater injectivity for deep reservoir penetration.

Both gel systems exhibited excellent plugging performance, achieving residual resistance factors (RRF) of 185 (rigid gel) and 93 (soft gel), significantly exceeding the industry benchmark of RRF > 20. These results confirm their effectiveness in reducing water permeability and resisting washout. With gelation times over 8 h and plugging efficiencies above 95%, the systems offer strong operational flexibility for field applications.

Rotational rheological analysis of gelling solutions highlighted the role of crosslinkers and ionic interactions in viscosity modulation. KCl reduced viscosity via electrostatic shielding, while covalent crosslinking strengthened gel structure, improving shear resistance and mechanical stability, making the system highly adaptable for water shut-off applications.

The dual-polymer gel maintained an elastic modulus of ~7.2 Pa up to 21.5% strain, with G′ about 3.67 times higher than G″, indicating strong mechanical integrity for near-wellbore applications. In contrast, the soft gel, with an elastic modulus 2.58 times lower than the rigid gel, remained stable up to 90% strain and could stretch up to 900% before structural breakdown. These results demonstrate that both gels effectively reduce permeability while maintaining their properties under high-strain conditions in the reservoir.

## Figures and Tables

**Table 1:** Composition and properties of synthetic brine.

Ionic Composition	Cations		Anions		Salinity
	Na^+^ y K^+^	Ca^+2^	Cl^−^	HCO_3_^−^	
Concentration (ppm)	327.4	6.9	180	525	1039.3

**Table 2:** Sandpack mineral composition.

Mineral	Quartz	Feldspars	Shale	Siderite
Mass fraction (%)	74	2	23	1

**Table 3:** Sydansk code. Adapted from [22].

Code	Description
A	Non-detecting gel: The viscosity of the system is equivalent to the viscosity of the polymer, and the formation of a gel cannot be observed with the naked eye.
B	High flow gel: The viscosity of the gel system is slightly higher than the viscosity of the polymer.
C	Flowing gel: Most of the gel flows to the top of the vial after inversion.
D	Medium Flow gel: When the glass bottle is turned over, a small amount of gel (mass fraction <15%) cannot flow to the other end, often in the shape of a tongue.
E	Hardly flowing gel: When the glass bottle is turned over, a few gels can slowly flow to the other end, and most of them (mass fraction >15%) are not fluid.
F	High deformation nonflowing gel: The gel cannot flow into the mouth of the bottle when the glass bottle is turned over.
G	Medium deformation nonflowing gel: When turned over, it can only flow to the middle of the glass bottle.
H	Slightly deformed nonflowing gel: When flipped, only the surface of the gel is deformed.
I	Rigid gel: When turned over, the surface of the gel does not deform.
J	Ringing gel: When shaking the glass bottle, you can feel the mechanical vibration like a tuning fork.

**Table 4:** Final strengthening and initial gelation time for rigid gel.

HPAM: PEI→ HPAM Concentration↓	1:1		2:1		4:1	
	Gelation Time (h)	Strength Code	Gelation Time (h)	Strength Code	Gelation Time (h)	Strength Code
10,000 ppm (5000 ppm HMW and 5000 ppm LMW)	4	H	6	G	10	G
15,000 (5000 ppm HMW and 10,000 ppm LMW)	4	H	5	G	9	G

**Table 5:** Final strengthening and initial gelation time for soft gel.

Polymer Concentration (ppm)	Floret AN9-901 (PM = 7 × 106 Dalton)	
	Gelation Time (Hours)	Strength Code
2000	>24	C
4000	13	D
5000	11	E
6000	10	F
8000	9	G

## Data Availability

The original contributions presented in this study are included in the article. Further inquiries can be directed to the corresponding author.
